# Infographic: Injuries in Sail GP Season 4

**DOI:** 10.1177/19417381251408607

**Published:** 2026-01-28

**Authors:** Thomas Fallon, Helene Rousselon, Jon Deakin, Maria Fernandez, Neil Heron

**Affiliations:** † Centre for Public Health, Queen’s University Belfast, Northern Ireland; ‡ Edinburgh Sports Medicine Research Network and UK Collaborating Centre on Injury and Illness Prevention in Sport (UKCCIIS), Institute for Sport, PE and Health Sciences, University of Edinburgh, Edinburgh, UK; § Medical Department, SailGP, London, UK; ‖ British Columbia Emergency Health Services, Vancouver, Canada; ¶ School of Health Sciences, Justice Institute of British Columbia, New Westminster, Canada

**Keywords:** illness epidemiology, injury epidemiology, sailing

## Abstract

This infographic summarizes the first prospective injury and illness surveillance study in Sail Grand Prix (SailGP) during Season 4 (2023-2024). Monitoring 100 professional athletes across 4919.84 sailing hours, the study found an overall injury rate of 9.96 per 1000 hours—higher during racing (11.89) and particularly during foiling activities (26.52). Lower limb injuries, especially to the ankle and knee, were most common, with grinders showing the highest positional injury incidence. Illness, mainly respiratory in nature, occurred at 5.14 per 1000 hours. These findings highlight the physical demands and health risks of elite foiling, informing targeted prevention, conditioning, and safety strategies in SailGP.

Sailing is a dynamic and multifaceted sport that entails the navigation of boats using sails, with a worldwide participation of approximately 16 million people.^3^ Sailing encompasses diverse disciplines, ranging from Olympic classes to commercial racing, each presenting unique challenges that test the limits of human performance and resilience. Sail Grand Prix (SailGP), inaugurated in 2019, represents a cutting-edge evolution in elite sailing, characterized by high-speed, high-risk racing in F50 foiling catamarans. These technologically advanced vessels, capable of exceeding 90 kph, demand extreme physical, technical, and cognitive performance from athletes. However, the dynamic and high-intensity nature of SailGP also introduces distinct health risks, particularly musculoskeletal injuries and overuse syndromes.

Despite these challenges, epidemiological data in sailing, especially in foiling disciplines, has historically been limited. As with any high-performance sport, the fast-paced, worldwide environment of SailGP exposes athletes to significant risks of injury and illness.^6^ Understanding the epidemiology of these injuries and illnesses is crucial for developing effective prevention strategies.^2,7^ Injury and illness epidemiology investigates the distribution, determinants, and outcomes of health-related events within a defined population. To address this gap, the first prospective injury and illness surveillance study in SailGP was conducted during Season 4 (2023-2024), offering new insights into injury patterns, mechanisms, and incidence rates in this elite sporting environment.^2^

Across the season, 100 professional SailGP athletes were monitored, providing a combined exposure of 4919.84 hours of sailing, comprising both racing and training activities.^1^ The overall injury incidence was 9.96 per 1000 hours, with significantly higher rates observed during racing (11.89 of 1000 hours) compared with training (8.41 of 1000 hours). This differential highlights the increased biomechanical stress, and situational hazards present during competitive regattas, including abrupt maneuvers, high-impact water contact, and limited recovery opportunities. Notably, foiling-specific activity central to SailGP racing was associated with an even higher injury rate of 26.52 per 1000 hours, underscoring the unique physical demands imposed by this new racing format.

Anatomically, the lower limbs, particularly the ankle and knee, were the regions most affected. These injuries are consistent with the rapid directional shifts and stability demands inherent in foiling. Among onboard roles, grinders tasked with high-repetition, force-intensive tasks such as winching exhibited the highest positional injury incidence, at 3.86 injuries per 1000 hours. This reflects the cumulative load and restricted recovery associated with their responsibilities during both training and competition. Illness incidence was also recorded, with respiratory infections dominating and an overall illness rate of 5.14 per 1000 hours, likely influenced by extensive international travel, changing environmental conditions, and close-contact team environments.^4,5^


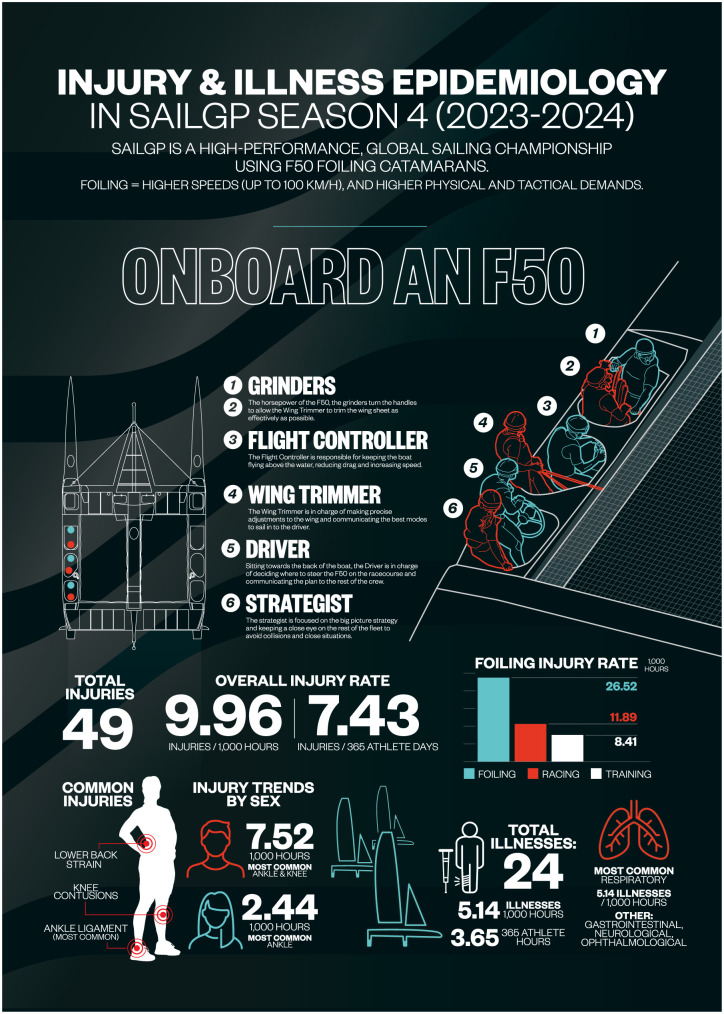


SailGP has specific boat simulators for training athletes to use the boats, helping to improve familiarity with these new boats, particularly around activities such as foiling, which are higher risk with the higher speeds involved. It would also be prudent to consider developing robust injury prevention routines specific to Sail GP and to develop these programs from the injury/illness epidemiology work. These programs could be developed similar to FIFA11+ GAA 15 and Prep-to-Play PRO injury prevention programs, which have been shown to reduce injury risk in these specific sports. Such programs have been shown to have significant reductions in injury incidence, which would have some crossover for Sail GP given the high injury rates seen in athletes, predominantly in the lower limb.

The findings from Season 4 establish a formative evidence base for medical teams, performance staff, and governing bodies. By identifying where, when, and how injuries are most likely to occur, these data support the development of targeted injury prevention strategies, role-specific conditioning, and load management protocols.^7^ Moreover, the successful implementation of a prospective surveillance model in SailGP demonstrates the feasibility of embedding structured health monitoring in fast-paced, elite-level sailing competitions. The infographic that follows visualizes these findings, highlighting key patterns in injury incidence, affected anatomical areas, and risk differentials across activities and crew roles, ultimately supporting a proactive approach to athlete safety and performance sustainability in professional sailing.
